# Large scale transcriptome analysis reveals interplay between development of forest trees and a beneficial mycorrhiza helper bacterium

**DOI:** 10.1186/s12864-015-1856-y

**Published:** 2015-09-02

**Authors:** Florence Kurth, Lasse Feldhahn, Markus Bönn, Sylvie Herrmann, François Buscot, Mika T. Tarkka

**Affiliations:** UFZ - Helmholtz Centre for Environmental Research, Department of Soil Ecology, Theodor-Lieser-Str. 4, 06120 Halle/Saale, Germany; UFZ - Helmholtz Centre for Environmental Research, Department of Community Ecology, Theodor-Lieser-Str. 4, 06120 Halle/Saale, Germany; German Centre for Integrative Biodiversity Research (iDiv) Halle – Jena – Leipzig, Deutscher Platz 5, 04103 Leipzig, Germany

**Keywords:** Plant growth promoting rhizobacteria (PGPR), Defence, Disease resistance, Mycorrhiza

## Abstract

**Background:**

Pedunculate oak, *Quercus robur* is an abundant forest tree species that hosts a large and diverse community of beneficial ectomycorrhizal fungi (EMFs), whereby ectomycorrhiza (EM) formation is stimulated by mycorrhiza helper bacteria such as *Streptomyces* sp. AcH 505. Oaks typically grow rhythmically, with alternating root flushes (RFs) and shoot flushes (SFs). We explored the poorly understood mechanisms by which oaks integrate signals induced by their beneficial microbes and endogenous rhythmic growth at the level of gene expression. To this end, we compared transcript profiles of oak microcuttings at RF and SF during interactions with AcH 505 alone and in combination with the basidiomycetous EMF *Piloderma croceum.*

**Results:**

The local root and distal leaf responses to the microorganisms differed substantially. More genes involved in the recognition of bacteria and fungi, defence and cell wall remodelling related transcription factors (TFs) were differentially expressed in the roots than in the leaves of oaks. In addition, interaction with AcH 505 and *P. croceum* affected the expression of a higher number of genes during SF than during RF, including AcH 505 elicited defence response, which was attenuated by co-inoculation with *P. croceum* in the roots during SF. Genes encoding leucine-rich receptor-like kinases (LRR-RLKs) and proteins (LRR-RLPs), LRR containing defence response regulators, TFs from bZIP, ERF and WRKY families, xyloglucan cell wall transglycolases/hydrolases and exordium proteins were differentially expressed in both roots and leaves of plants treated with AcH 505. Only few genes, including specific RLKs and TFs, were induced in both AcH 505 and co-inoculation treatments.

**Conclusion:**

Treatment with AcH 505 induces and maintains the expression levels of signalling genes encoding candidate receptor protein kinases and TFs and leads to differential expression of cell wall modification related genes in pedunculate oak microcuttings. Local gene expression response to AcH 505 alone and in combination with *P. croceum* are more pronounced when roots are in resting stages, possibly due to the fact that non growing roots re-direct their activity towards plant defence rather than growth.

**Electronic supplementary material:**

The online version of this article (doi:10.1186/s12864-015-1856-y) contains supplementary material, which is available to authorized users.

## Background

Soil microbial communities influence numerous physiological processes and traits of plants, including: seed germination, seedling vigour, growth and development, nutrition and the progression of various diseases [reviewed in 1]. Mutualistic fungi form close associations with plants that provide multiple benefits to both sets of organisms. Most temperate forest trees develop a mutualistic root symbiosis, ectomycorrhiza (EM), with fungi. This enhances the tree nutrient acquisition and the fungus ability to extend mycelia through the soil and form fruiting bodies [2]. Various other beneficial microorganisms are also frequently associated with EM, including mycorrhiza helper bacteria (MHBs) [3]. Production of bacterial secondary metabolites such as auxofuran [4] stimulate mycelial growth, but mycorrhiza formation can also be promoted by the reduction of soil-mediated stress [5, 6] and by augmentation of plant-fungus contacts by stimulation of lateral root formation [7]. Helper activities can also improve mycorrhizal functioning, for instance by mobilising nutrients from soil minerals [8] and protecting plants against attack by root pathogens [9]. Although the importance of EM-associated bacteria for the symbiosis is established, it is unclear how plants coordinate their gene expression responses to the MHB in the presence and absence of the EMF.

Like many tropical and some temperate trees, oaks display an endogenous rhythmic growth pattern with alternating phases of development and rest throughout their vegetative growth periods [10]. The endogenic character of rhythmic growth is reflected by the regular period of alternating shoot flushes (SF) and root flushes (RF) under uniform and constant environmental conditions [11–13]. It has been suggested that the rhythmic development is a consequence of carbon partitioning [14–17]. Herrmann, et al. [18] developed a microcosm system in which microcuttings of the pedunculate oak *Quercus robur* clone DF159 express these typical alternating flushes and using the same system, Angay, et al. [19] showed that concentrations of high nonstructural carbohydrates in roots are high during RF and low during SF.

Rhythmic growth of oak trees affects their interactions with associated microorganisms. For instance the distribution pattern on the mother roots and the frequency of mycorrhizal root tips are affected by the endogenous rhythmic growth of *Q. robur* [10]. In addition, infestation of roots by the oomycete pathogen *Phytophthora quercina* is enhanced during RF, when roots are growing rapidly and the plant below-ground carbon allocation is highest [19], and colonisation of leaves by oak powdery mildew is enhanced during SF, when the leaves are growing most rapidly [20]. Oaks are a group of major broadleaf forest trees in Europe that are key components of complex networks of biotrophic interactions, involving relationships with endophytic [21], pathogenic [22] and EM [23] fungi, as well as bacteria that stimulate EM formation [24]. Since the impact of rhythmic growth on biotic interactions of the oak is so evident, it should be investigated if it affects the gene expression response of the oak to these interacting organisms.

Previously, we showed that co-inoculation of oak microcuttings with the mycorrhiza helper bacterium *Streptomyces* sp. strain AcH 505 (hereafter AcH 505) led to an increase in the number of *Piloderma croceum*-mycorrhizal plants [25]. Furthermore, AcH 505 induced a systemic defence response against *Microsphaera alphitoides* in pedunculate oak leaves, involving both jasmonic acid (JA)/ethylene (ET) and salicylic acid (SA)-dependent signalling [26]. However, the cited studies did not investigate variations in transcriptomic responses to AcH 505 during RF and SF growth stages, effects of AcH 505-treatment in roots, or its interactions with mycorrhizal fungi, which are crucial elements of a MHB functional role in the oak system.

EMFs, such as *P. croceum* are essential for optimal development of pedunculate oak microcuttings [23, 27]. Extensive re-programming of the oak transcriptome has been detected both during pre-symbiotic development and in mature symbiotic EM with *P. croceum* [28–30]. For instance, EM formation with *P. croceum* leads to defence suppression in oak, embodied by low abundance of defence-related transcripts [30]. But the influence of dual presence of AcH 505 and *P. croceum* on the oak was not investigated.

When microorganisms interact with plants, perception processes play a crucial role. Plants perceive microorganisms by sensing microbe-associated molecular patterns [MAMPs; 31]. MAMP receptors of plants are encoded by *R* genes including transmembrane receptor-like kinases (RLKs), receptor-like proteins (RLPs) and leucine rich repeat-nucleotide binding (LRR-NB) proteins [32] and they can recognize both pathogenic [33] and beneficial microorganisms [31]. Transcription factors (TFs) of the APETALA 2/ethylene-responsive family, in particular ethylene-responsive element binding factors (ERFs) play a major role in integrating the response to biotic interactions and are implicated in MAMP-induced systemic resistance signalling [34]. Seven ERF contigs showed up-regulation in *P. croceum*/oak EM [30], but it has not been investigated how AcH 505 treatment affects their transcription.

Based on our analysis of systemic defence response in leaves to AcH 505 [26] and on the defence suppressing effect of *P. croceum* on oaks [30] we questioned i) how the oak coordinates its gene expression responses to MHB in the absence and presence of the EMF, ii) how the gene expression response of the oak to the interacting organisms is affected by the stage of growth, iii) if AcH 505 affects the *P. croceum* attenuated defence related gene expression in oak or vice versa, and iv) if AcH 505 treatment affects the transcription of ERFs. This work was based on two hypotheses. Our first hypothesis states that AcH 505 induces, while co-inoculation with *P. croceum* attenuates the defence responses in oak. Our second hypothesis states that local molecular responses to the organisms interacting with their roots are stronger during SF, expressed as differential representation of *R* genes, hormonal signalling genes and cell wall proteins. The second hypothesis is based on the observations of Angay, et al. [19] that oak roots express a higher level of root colonisation by *P. quercina* in RF, and also, on the general literature on MAMP perception, hormonal signalling and cell wall modifications occurring during plant-microbe interactions [e.g. 33, 34]. To address these hypotheses, we investigated transcriptional alterations in oak during the colonisation by AcH 505 and the co-colonisation with *P. croceum*, focussing on the responses of roots and leaves during RF and SF. The gene expression data demonstrates that the transcriptional response of the oak is more active during SF, the period when the roots are not growing, that a defence response is induced by the mycorrhization helper bacterium but attenuated by the EMF, and that several candidate genes related to MAMP recognition processes and control of transcription are differentially expressed (DE) upon these treatments.

## Results

**Fig. 1:**
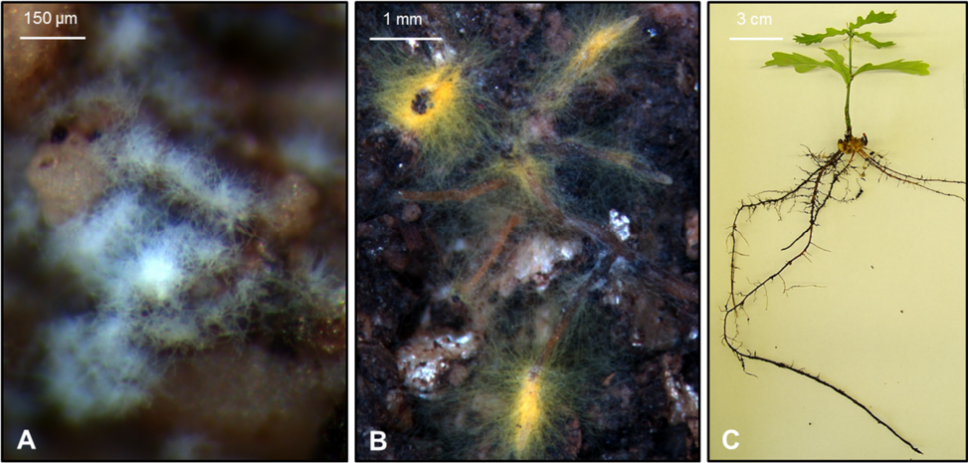
Pedunculate oak microcuttings with interacting microorganisms. *Streptomyces* sp. AcH 505 on soil particles in the microcosm (**a**) *Piloderma croceum* – oak ectomycorrhizas (**b**) and a pedunculate oak *Quercus robur* microcutting (**c**)

**Fig. 2:**
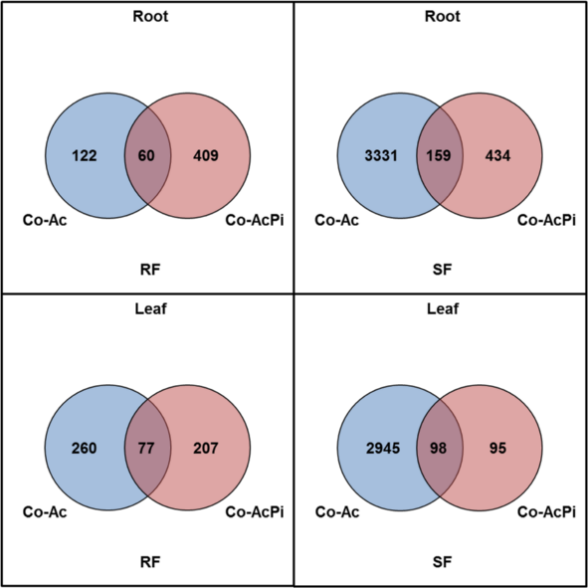
Venn-diagrams illustrating numbers of DECs (Benjamini-Hochberg adjusted, p ≤ 0.01). The comparisons depicted are for the following pairs of roots and leaves of microcuttings during RF and SF: Control versus AcH 505 inoculated (Co-Ac) and Control- versus AcH 505- and *P. croceum-*inoculated (Co-AcPi)

Oak microcuttings were successfully inoculated with AcH 505 (Fig. 1). Three replicate RNA-Seq datasets were generated for microcutting lateral roots and leaves, during both RF and SF, following no inoculation, inoculation with AcH 505 and with both AcH 505 and *P. croceum* (*n* = 36; Additional files 1 and 2). For differential gene expression analysis, pairwise comparisons of datasets from plants inoculated with AcH 505 versus non-inoculated plants [differentially expressed contigs (DECs) in AcH 505 inoculated plants] and plants inoculated with AcH 505 and *P. croceum* versus non-inoculated plants [DECs in co-inoculated plants] were performed for roots and leaves at RF and SF respectively. The validity of the differential expression analyses was confirmed by qRT-PCR analysis (Additional file 3). Numbers of DECs among the samples are visualised in the Venn diagrams shown in Fig. 2 and tabulated in Additional file 4. AcH 505 treatment induced more DECs during SF than during RF in both roots and leaves. A suppressive effect by AcH 505-*P. croceum* co-inoculation on the numbers of DECs was evident during SF. Proportions of contigs regulated in AcH 505 inoculated plants which were also regulated when subjected to co-inoculation with *P. croceum*, were 33 in roots and 23 % in leaves during RF, and 5 and 3 % during SF.

**Fig. 3:**
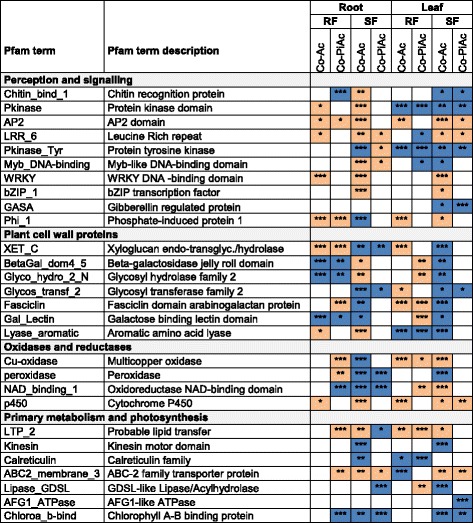
Protein family (Pfam) terms enriched for up- and down-regulated contigs, in roots and leaves harvested from plants during RF and SF, treated with AcH 505 (Co-Ac) and both microorganisms (Co-AcPi). Putative function categories “perception and signalling”, “plant cell wall proteins”, “oxidases and reductases” and “primary metabolism and photosynthesis” are given. Orange colour indicates up-regulated and blue down-regulated enriched Pfam terms. Significance levels are marked by asterisks in the boxes (*** :p ≤ 0.001; **: 0.001 < p ≤ 0.01; *: 0.01 < p ≤ 0.05). The p-value cut-off was set at p ≤ 0.05

**Fig. 4:**
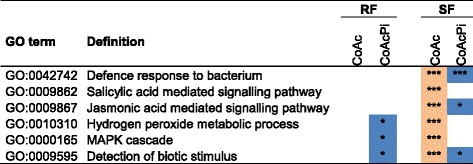
Enriched plant defence-related GO terms in pedunculate oak roots, showing terms enriched for up-regulated contigs after AcH 505 inoculation specifically during SF and partially depleted in co-inoculated plants. GO enrichment analysis was implemented by GOseq. Orange colour indicates up-regulated and blue down-regulated enriched Pfam terms. Significance levels are marked by asterisks in the boxes. (*** :p ≤ 0.001; **: 0.001 < p ≤ 0.01; *: 0.01 < p ≤ 0.05). The *p*-value cut-off was set at *p* ≤ 0.05

To obtain a comprehensive view of differential gene expression, we conducted functional annotation enrichment analyses with GOseq. The 10 most significantly enriched terms in Protein family (Pfam) and Gene Ontology (GO) categories are shown in Additional file 5. Representative enriched Pfam terms are shown in Fig. 3, enriched plant defence-related GO terms in roots in Fig. 4, and representative DECs for the Pfam and GO terms, as well as plant defence-related DECs in roots, are listed in Additional file 6.

Pfam enrichment analysis revealed significant changes in Pfam terms related to perception and signalling, plant cell wall proteins, oxidases and reductases, as well as primary metabolism and photosynthesis in both AcH 505- and co-inoculated plants (Fig. 3; Additional file 5). In roots at RF, plant cell wall protein related Pfam terms were strongly enriched. For instance, *Xyloglucan endo-transglycosylase* (*XET*) was enriched in up-regulated contigs (EUC) in both treatments and *Fasciclin domain arabinogalactan protein* (*FDA*) in co-inoculated plants, with other EUC including *Phosphate-induced protein 1 conserved region* (*PIP1*) and *Probable lipid transfer* (*LTP*). The Pfam term EUC was associated with increased abundance of several predicted XET, PIP1, FDA and LTP transcripts (Additional file 6). The terms which were enriched in down-regulated contigs (EDC) included *Beta-galactosidase* and *Galactose binding lectin domain* in AcH 505 treatment, but photosynthesis and plant defence related terms in co-inoculation, with Pfam terms *Chlorophyll A-B binding protein* (CHLBP) and *Chitin recognition protein*. In roots at SF under AcH 505 treatment, the most significant Pfam terms EUC were related to stress associated transcriptional regulation, such as *Bzip* and *WRKY DNA-binding domain,* the latter with seven up-regulated candidate *WRKY* contigs (Additional file 6). AcH 505 treatment led to Pfam terms EDC of the types *Multicopper oxidase* and *Glycosyl transferase family 2*, and co-inoculation the term *CHLBP* with 24 down-regulated candidate CHLBP contigs (Additional file 6), as well as the development and defence response related term *GDSL-like Lipase/Acylhydrolase (GDSL).* In leaves at RF, the terms EUC included *PIP1* and *XET* with associated contigs (Additional file 6) in AcH 505 treatment, and *LTP* and *Galactose binding lectin domain* in co-inoculation. The terms EDC included *Protein kinase domain* and *Calreticulin* in both treatments and *ABC-2 transporter* in AcH 505 treatment. In leaves at SF, the terms EUC included *Multicopper oxidase* in AcH 505 treatment, and *GDSL* in co-inoculation*.* In contrast, the terms EDC included *Kinesin motor domain* and *GDSL* in AcH 505 treatment, and *Gibberellin regulated protein* and *AFG1-like ATPase* in co-inoculation.

AcH 505 inoculation led to GO term enrichment which corroborated qualitatively with Pfam term results (Additional file 5). For instance in roots at RF, plant cell wall GO term *Xyloglucan:xyloglucosyl transferase activity* was EUC in both treatments in corroboration with the Pfam term *XET*, and *Lipid transport* in co-inoculation, in line with the Pfam term *LTP*. Plant defence associated GO terms were prominently EUC upon AcH 505 treatment in roots, and when the oaks were co-inoculated with the EMF, these GO-terms were EDC or non-significant (Fig. 4). Among the contigs which were up-regulated upon AcH 505 but not by co-inoculation, we detected disease resistance, protein kinase, TF, and chitinase contigs (Additional file 6), indicating that suppression of plant defence by *P. croceum* [30] overrules the defence-stimulating effect of AcH 505 in roots.

To address the AcH 505 driven gene expression in more detail, we searched for those individual contigs that were regulated as well in AcH 505 as in co-inoculation (Table 1). We reasoned that those genes regulated in AcH 505 but not influenced by additional presence of *P. croceum,* may be part of the core interactome gene set of AcH 505 on the oak. In the roots of plants during RF, these core contigs were related to plant growth (XET) and signalling (*Kinase-like protein*). During SF, the core contigs were related to transcriptional regulation and redox reactions (*Chromatin binding protein, Peroxidase*), and the down-regulated contigs included photosynthesis related *PS II binding protein LHCB1.5*. In the leaves of the microcuttings, based on the *Arabidopsis thaliana* orthologs, the contigs with corresponding patterns identified during RF were related to cell wall biosynthesis (*Cellulose synthase, Fasciclin-like arabinogalactan-protein*), lipid transport or signalling (*Lipid transfer protein*), and transport (*PHO1 phosphate exporter*), whilst during SF the core contigs were involved in defence and growth-related signalling (*Calcium-binding protein, LRR nucleotide binding sequence [NBS] resistance protein, anthocyanidin synthase*).

**Table 1:** Contigs regulated in AcH 505 inoculated plants which were not altered when subjected to co-inoculation with *P. croceum* in roots and leaves and during RF and SF

	Co-Ac		Co-PiAc		
Contig	Log_2_ fold change	FDR	Log_2_ fold change	FDR	Sequence description
	Root – RF				
comp34428_c0_seq2	8,8	2,0E-07	7,7	1,3E-04	Ice binding
comp43120_c0_seq11	2,6	7,4E-07	2,2	6,6E-17	Xyloglucan endotransgl/hydrolase
comp36512_c0_seq1	5,9	1,1E-06	6,0	5,3E-08	Nucleolar complex protein
comp32163_c0_seq1	2,2	1,3E-05	2,9	8,3E-09	Clavaminate synthase
comp43753_c1_seq2	8,6	9,0E-05	8,6	3,9E-08	Kinase-like protein
comp39841_c1_seq1	1,1	6,0E-05	1,1	2,0E-06	Exordium-like protein
comp37819_c1_seq5	7,9	1,5E-03	8,5	3,6E-10	o-linked c transferase
comp43557_c1_seq2	8,4	7,0E-03	8,6	2,2E-10	tRNA-dihydrouridine synthase
	Root – SF				
comp29599_c0_seq1	−1,3	6,6E-19	−1,9	9,9E-03	Gibberellin-regulated protein
comp41819_c4_seq2	−8,5	1,3E-10	−8,7	4,5E-07	F-box family protein
comp43426_c1_seq15	−7,2	4,8E-07	−7,4	1,9E-04	Sugar transporter
comp37704_c0_seq2	−1,0	3,1E-04	−3,9	5,5E-44	PS II binding protein LHCB1.5
comp42662_c0_seq8	1,6	1,1E-99	1,1	3,1E-05	Cytochrome p450
comp19461_c0_seq1	1,2	7,0E-54	1,1	1,4E-04	Peroxidase 10
comp35114_c0_seq1	1,1	1,4E-45	1,4	5,4E-04	Zinc finger protein
comp19664_c0_seq1	0,8	2,2E-11	1,0	2,3E-03	Thaumatin
comp42100_c0_seq10	7,2	9,2E-06	6,9	7,8E-04	Chromatin binding
	Leaf – RF				
comp36279_c0_seq4	−9,0	1,1E-09	−8,9	1,9E-09	GTP cyclohydrolase
comp23339_c0_seq1	−1,5	1,6E-06	−1,8	7,6E-10	Phosphoglycerate dehydrogenase
comp40475_c2_seq3	−1,1	8,2E-04	−0,9	3,0E-03	Leucine-rich repeat receptor
comp28232_c0_seq1	2,3	4,6E-04	2,0	5,4E-04	Fasciclin-like arabinogalactan-protein
comp30075_c0_seq1	1,8	4,8E-03	2,2	1,4E-05	Lipid transfer protein
comp42008_c3_seq5	3,7	3,5E-06	3,1	2,4E-03	Calcium-binding protein
comp39615_c0_seq1	2,2	3,9E-06	1,8	9,3E-04	Multicopper oxidase
comp42669_c0_seq2	5,1	1,2E-05	3,8	6,2E-03	Cellulose synthase
comp42379_c0_seq7	4,3	9,0E-03	4,4	2,1E-03	Pho1-like protein
	Leaf – SF				
comp30731_c0_seq2	−0,7	1,9E-20	−0,9	1,3E-03	Cbl-interacting serine threonine-protein
comp30800_c0_seq1	−2,7	1,8E-06	−2,2	3,6E-03	Adenylyl-sulfate reductase
comp39235_c1_seq5	−6,7	1,8E-04	−6,7	5,8E-03	Chaperone protein
comp21202_c0_seq1	3,4	3,4E-85	1,4	1,7E-04	Anthocyanidin synthase
comp42008_c3_seq5	2,5	1,6E-41	2,6	6,0E-04	Calcium-binding protein
comp42290_c0_seq1	1,2	3,6E-27	1,8	9,2E-10	Lipoxygenase
comp42379_c0_seq7	8,6	5,4E-11	9,0	1,2E-03	Pho1-like protein
comp43826_c1_seq6	8,8	4,1E-10	7,9	1,1E-05	LRR-NB-ARC domain protein

Significant differential expression was determined by edgeR with a threshold Benjamini-Hochberg adjusted p-value of 0.01, indicated by "FDR"

Abbreviations: endotransgl, endotransglycosidase; PSII, photosystem II

Due to the Pfam term enrichment in both treatments, both stages of growth, and tissues, the Leucine Rich Repeat (LRR) and Ethylene response transcription factor (AP2/ERF)-associated DECs were treated in more detail and their homology to orthologous transcripts in *A. thaliana* was estimated by blastx analysis (Additional file 6). Most of the predicted LRR contigs encoded members of the extensive families of LRR receptor-like kinases (LRR-RLKs) and receptor-like proteins (LRR-RLPs) with roles in *A. thaliana* ranging from recognition of microorganisms to signalling in growth and differentiation. The dominance of SF vs. RF in the numbers of DECs was particularly strong for the predicted LRR containing defence response regulators. From the 34 DECs of this type, 31 occurred at SF but only 3 at RF (Additional file 6). These DECs comprised candidate leucine-rich repeat-nucleotide binding site (LRR-NBS) contigs, which act as *R* genes in *A. thaliana*. Predicted functions of other identified LRR-encoding DECs are related to auxin responses, microtubule cytoskeleton, cell wall composition and terpenoid biosynthesis. Among these signalling devices and TFs, which are most important for the AcH 505 interaction, contigs regulated in both AcH 505- and co-inoculations were detected. From LRR-receptor protein kinase encoding contigs, a contig (homologous to *A. thaliana AT3G47110*) was up-regulated in leaves, and another (*AT3G14840*), was down-regulated in leaves at RF and SF by both treatments. Contig encoding LRR-NB-ARC domain protein (*AT3G14460*) was up-regulated in both roots and leaves at SF. From TFs, AP2/ERF contig homologous to *A. thaliana SHN2* (*AT5G11190*) was up-regulated in roots and leaves at RF by both treatments, and by AcH 505 at SF (Additional file 6). Among the co-inoculation treatment specific DECs, i.e. contigs which were not DE by sole AcH 505 treatment, we identified contigs homologous to *A. thaliana* LRR-RLP gene *AtRLP9*, LRR-RLK gene *AtCLV1* and TIR-NB-LRR domain protein gene *AtTAO1* (Additional file 6).

## Discussion

The RNA-Seq approach has been previously used to explore both global transcriptomic responses of oaks to microbes (mutualists and pathogens) [26, 30, 35]. In the study presented here, we applied RNA-Seq analysis to investigate the impact of the MHB AcH 505 on the oak during two rhythmic growth stages and used combined treatment with the EMF *P. croceum* to disentangle the direct impact of AcH 505 from the one that is modified in case of co-inoculation. Strong response of the microcutting roots and leaves to the AcH 505 treatment was observed at the root-resting stage. It was manifested by a high number of DECs, and it was in part attenuated by the *P. croceum* treatment. We began to unravel the gene expression networks linking the microcutting perception of AcH 505 to the plant development, and detected a candidate plant immune response network in the roots following the bacterial inoculation. In this way, our results provide foundation for comparative analyses of interactions between oaks and other microorganisms to elucidate fundamental patterns.

### Perception of the microorganisms in oak microcuttings

In accordance with our first hypothesis, we found that defence gene expression in the oak microcuttings were induced by AcH 505, but attenuated by co-inoculation with *P. croceum* (Fig. 4; Additional file 6). This suggests that the defence suppression by *P. croceum* dominates over the elicitation of defences by AcH 505. For instance, contigs encoding NDR1-like disease resistance protein, chitinases and hevein were up-regulated genes upon AcH 505 inoculation, but not so upon co-inoculation (Additional file 6), and the GO-term *Jasmonic acid mediated signalling pathway* was EUC upon AcH 505 inoculation but EUD upon co-inoculation (Fig. 4). The only thus far characterised mechanism how an EMF suppresses plant defences has been presented by Plett, et al. [36]. They showed that *Laccaria bicolour* produces an effector, mycorrhiza-induced small secreted protein 7 (LbMISSP7) that promotes mycorrhiza formation by blocking plant defence-related JA signalling. *P. croceum* expresses mycorrhiza-induced small secreted proteins (MISSPs) during EM formation with oak (F. B., S. H., M. T., unpublished), but none of the *P. croceum* MISSP genes have been functionally characterized. Nevertheless, the suppression by *P. croceum* observed in co-inoculated plants seems to be a local effect, as it was only detected in roots. Accordingly, Mailänder [20] showed that inoculation of plants with *P. croceum* did not facilitate powdery mildew infection of oak leaves. In contrast, the enhanced defence gene expression in AcH 505-inoculated plants was also observed in the leaves, suggesting a systemic effect. This supports our previous finding that inoculating pedunculate oak roots with AcH 505 elicits a defence response against *M. alphitoides* in the leaves [26].

In accordance with our second hypothesis, the gene expression data demonstrated that the transcriptional response of the oak is more active during SF, the period when the roots are not growing. The oak gene expression response to AcH 505 in SF includes DECs encoding resistance proteins, involved in pathogen recognition and subsequent activation of innate immune responses. Candidate LRR containing defence response regulator genes had a particularly SF-specific gene expression (Additional file 6). The strongest induction, 16 to 70-fold expression levels in SF roots and leaves upon AcH 505 and co-treatment, was observed for the *LRR-NB-ARC domain protein* contig with close homology to *AT3G14460* (Table 1). *AT3G14460* is a putative disease resistance gene in *A. thaliana*, and functional characterisation of the oak gene would now prove its role in the recognition of AcH 505.

Detailed analysis of the enriched LRR Pfam terms identified in our experiment in both treatments in roots and leaves during SF (Fig. 3) revealed differential expression of transmembrane RLKs and RLPs (Additional file 6). Some of the LRR-containing receptor transcripts identified in this study were DE in AcH 505- and co-inoculated oaks, suggesting a function as important core genes and central response regulators of the MHB. Among them, a homologue with implicated role in defence against pathogens in *A. thaliana* [37], an RLK contig homologous to *AT3G47110,* was strongly (up to 70-fold) up-regulated in roots at SF and at RF by both treatments. The induction at both stages of growth indicates that this candidate RLK may be an important core element of AcH 505 signalling. The strongest up-regulation (up to 80-fold) in leaves occurred for an RLK contig homologous to disease resistance family protein *AT2G34930*. The *AT2G34930* gene is induced in *A. thaliana* upon infection by the biotrophic oomycete downy mildew pathogen *Hyaloperonospora arabidopsidis* [37]. These strongly up-regulated transcripts we identified are probably connected to recognition events and maintenance of the interaction with AcH 505.

### Transcription factors in plant response to the microorganisms

Crucial part of the plant response to microorganisms and abiotic stressors is mediated by transcriptional control, which is implemented, in large extent, by three families of TFs: ethylene-responsive-element-binding factors (ERF), basic-domain leucine-zipper (bZIP) and WRKY proteins [38]. We presented evidence of DECs among members of these three TF families, occurring in the response of the oak to AcH 505 and/or co-inoculation (Additional file 6). A novel observation for the AcH 505-interaction was the identification of DE ERF contigs amongst the core transcriptome affected by both treatments (Additional file 6). In EM, ET signalling may be involved in control of the depth of colonisation of the plant apoplast by the EMF [39]. Our data indicates that as a MHB, AcH 505 may affect this process by the modification of ERF expression. For instance, *ERF8* contig was up-regulated in roots by the interaction with AcH 505 as well as by co-inoculation. Interestingly, the homologue in *A. thaliana, AtERF8* (*AT1G53170*) is a participant in a signalling node of three interacting ERFs, which appear to negatively regulate chitin signalling in defences against fungi, but positively regulate SA signalling in plant defences against the bacterial pathogen *Pseudomonas syringae* [40]. If the *ERF8* homolog of oak shares the functional properties of *AtERF8*, warrants further analysis.

In tobacco, AP2/ERF subfamily dehydration-responsive element-binding protein gene *DREB1B* overexpression confers improved abiotic and biotic stress tolerance [41] and in our analysis, the *DREB1B* expression increased in roots and leaves upon the treatment with AcH 505. In contrast, the gene expression data of Lesur, et al. [42] suggests that candidate DREB1A protein of *Q. robur* plays a role in the maintenance of bud dormancy, and it thus seems that the DREB subfamily of TFs deserves further attention in oaks.

In *A. thaliana*, a subgroup of bZIPs is involved in an abscisic acid (ABA) dependent signal transduction pathway under drought and high-salinity conditions, but also in biotic interactions [38]. The induction of oak bZIPs by AcH 505 treatment (Additional file 6) indicates a stress or defence response upon AcH 505 inoculation. The induced bZIPs encoded homologs of central ABA signalling associated TFs, in particular the predicted bZIP63 and GBF3 proteins. From these bZIPs, bZIP63 action relates sugar signalling to ABA synthesis and ABA sensitivity [43], whereas GBF3 is induced by ABA and plays a role in plant response to drought stress and ABA [44] in *A. thaliana.* We have previously observed that ABA defence pathway may play a role in AcH 505-based priming of host responses against powdery mildew infection [26], and the role of ABA mediated gene expression changes is supported by our data on light-harvesting chlorophyll a/b-binding protein (*LHCB*) expression as well. The LHCBs are apoproteins of the light-harvesting complexes of chloroplasts, and they are expressed ubiquitously in different plant tissues including roots [45]. Expression of the LHCB genes is modulated by various environmental and developmental cues, including the inducer light [46], and the repressors oxidative stress and ABA [47, 48]. The down-regulation of LHCB expression in roots can be interpreted as a stimulation of normal, darkness related down-regulation, but in leaves, the decrease in LHCB transcript levels suggests increased levels of stress, particularly upon AcH 505 inoculation. The current data further indicates that the suggested induction of stress by AcH 505 is attenuated by *P. croceum*, since none of the contigs associated with the bZIP category were induced, and only two out of of eleven AcH 505-suppressed LHCB contigs were suppressed upon co-inoculation in leaves at SF (Additional file 6). This raises the question as to how AcH 505, powdery mildew and *P. croceum* treatments would affect the response of oak to abiotic stress and affect ABA signalling.

Our third TF group of interest, the large family of WRKY proteins plays key roles mediating the regulation of plant defence and abiotic stress transcription and controlling gene expression at leaf senescence [38]. Among the AcH 505-induced WRKYs, homologs of three WRKYs which play roles in signal integration in *A. thaliana* were detected. WRKY70 protein activates SA-induced genes but represses JA-responsive genes, i.e. controls how the signals converge from these mutually antagonistic pathways [49], and WRKY50 proteins mediate SA-mediated repression of JA signalling [50]. The convergence of signalling pathways is also mediated by WRKY57 protein, which in *A. thaliana* is a repressor in JA-induced leaf senescence, and a common component of the JA- and auxin-mediated signalling pathways [51]. These results underline the urgency to characterise SA and hormonal signalling pathways in the oak system.

### Differential representation of transcripts related to growth and differentiation

The DECs identified suggest that AcH 505 treatment may support the processes of growth and differentiation in oak. Deeper analysis of the DE members of oak AP2/ERF subfamily (Additional file 6) identified a candidate SHINE clade of AP2 domain TF contig. The contig was induced by both treatments in roots and leaves. The corresponding gene *AtSHN2* in *A. thaliana* regulates wax biosynthesis, pectin metabolism and cell wall structure [52], and the induction of a *AtSHN2* homolog by both treatments in oak microcuttings might thus be related to the regulation of plant development. Changes in plant growth rates and patterns induced by bacteria and mycorrhizal fungi may be due to associated modifications of the main signalling pathways mediated by not only ET, but also auxin [53]. A homologue of *BAK1-interacting RLK1* gene (*BIR1, AT5G48380*), which stimulates the expression of auxin response regulators in *A. thaliana* [54] was induced by AcH 505 and a homologue of *A. thaliana AIR9* (*AT2G34680*) gene, an auxin-induced microtubule associated protein which is part of the mechanism positioning the direction of cell division [55], was induced by both AcH 505 and co-inoculation (Additional file 6).

Exordium (EXO) and Exordium-like genes encode cell wall proteins [56], and are structurally related to the tobacco *Phosphate-induced protein 1* gene, which is re-activated in cultured cells following release from cell cycle arrest caused by phosphate starvation [57]. Enriched Pfam term *PIP1* was associated with increased transcription levels of candidate EXO contigs in both AcH 505 and co-inoculation. In *A. thaliana*, the EXO proteins connect the extracellular carbon status to growth [58], regulate cell division activity in roots [59], and are essential for cell expansion in leaves [60]. In oak roots, the expression of EXO is upregulated at RF by AcH 505 and co-inoculation, suggesting that the treatments may affect cell division activity of oak roots.

The Pfam term *Xyloglucan endo-transglycosylase* (*XET*) was enriched for up-regulated contigs in RF in both treatments (Fig. 3), and for instance, one of the oak XET transcripts was induced by AcH 505 alone and together with *P. croceum* in RF roots (Table 1). In contrast, the Pfam term *XET* was enriched for down-regulated contigs in SF in both treatments (Fig. 3), and for instance, the oak XET transcripts with homology to the *AtXTH23* gene were down-regulated by both treatments in SF roots (Additional file 6). XET transcripts [61] and activities [62] are largely localized in the root elongation zone, and XET activity is required for the maintenance of root elongation [63]. Thus, since the RF developmental stage coincides with maximal root elongation rate [18], and SF with growth cessation, this association indicates that AcH 505 may enhance root elongation process in oaks during RF and suppress root elongation in SF.

Reversed pattern was observed for the Pfam term *Beta-galactosidase,* enriched for up-regulated contigs in SF in roots and RF in leaves, and enriched for down-regulated contigs in RF in roots and SF in leaves. The homology of the predicted oak proteins to plant beta galactosidases indicates that they may support tissue differentiation by the modification of cell wall galactans. Of the homologs, *A. thaliana BGAL12* (*AT4G26140*) is localised within cell walls [64], and associated with root differentiation [65]. This association suggests that AcH 505 might stimulate the maturation of cell walls in the rest stages. This is also supported by the induction of an oak peroxidase transcript by AcH 505 alone and together with *P. croceum* (Table 1). The oak peroxidase contig is orthologous to the product of *A. thaliana* gene *AtPER10* (*AT1G49570*), known to be up-regulated after ET treatment [66]. Increases in ET and peroxidase activities are both probably involved in the stimulation of peroxidase-mediated crosslinking in the cell wall, which prevents cell expansion [66]. Thus, during SF the up-regulation of peroxidases in oak roots may also contribute to cell wall maturation.

Together, these results suggest an interactive relationship between oak cell wall dynamics and plant-AcH 505 interactions. A similar connection has been suggested for mycorrhizal symbioses in oaks [30, 35] and *Populus* [39]. Detailed light and electron microscopical analyses of the suppression of Norway spruce root infection by the fungal pathogen *Heterobasidion abietinum* by the plant-protecting bacterium *Streptomyces* strain GB 4–2 [67] revealed how the cell walls were reinforced in the lateral roots of co-inoculated plants, preventing the colonisation of the spruce vascular cylinder by the fungal pathogen. In contrast, upon *Streptomyces* AcH 505-*Heterobasidion abietinum*-co-inoculation of Norway spruce, the lateral root cell wall structures were not altered [68], and root anatomical analyses were therefore not included in this work. But the current gene expression data indicates that in the oak system, cell biological analysis of the AcH 505-*P croceum*-interaction is also warranted.

## Conclusions

Rhythmic growth affected the gene expression response of oak to the interacting organisms, with much weaker molecular response in RF in terms of the number of DECs. This might indicate that the physiology in roots is rather devoted to growth at RF while the processes directed towards interactions are in most part attenuated. Future work will show if the repressed gene expression response consequently interferes with elicitation of defence responses or symbiosis related signalling in RF. Only few contigs were DE by both AcH 505 and co-inoculation, indicating that oak coordinates its gene expression responses to AcH 505 in the presence and absence of the EMF by induction of a few, specific MAMP receptors and TFs, i.e. candidate “core genes” of AcH 505 response. Our gene expression data further indicate a pivotal role of the cell wall as the responsive element in responses to AcH 505 alone and during co-inoculation with the EMF. Future work needs to focus on the identified candidate genes to verify their involvement in these processes. Clearly, there is also an urgent need to associate these oak genes with hormonal signalling pathways, interactions with insects and pathogens, and with the impact of abiotic environment.

## Methods

### The soil-based culture system

The pedunculate oak clone DF159 (*Quercus robur* L*.*) was micropropagated and rooted according to Herrmann, et al. [69], then cultivated in soil-based microcosms by placing rooted microcuttings in Petri dishes filled with gamma-sterilized soil as previously described [30]. The conditions for bacterial and fungal cultivation and plant inoculation were as described by Tarkka, et al. [30]. Briefly, *Piloderma croceum* J. Erikss. & Hjortst. Strain 729 (DSM- 4824) inoculum was produced by inoculating a substrate mixture of vermiculite and sphagnum peat with a 2-week-old liquid fungal culture. It was inoculated at establishment of the microcosm by mixing it 1:1 (v/v) with the gamma-sterilized soil. *Streptomyces* sp. AcH 505 was originally isolated from the soil around Norway spruce mycorrhizas in Haigerloch, Germany [70] and maintained on ISP2 agar medium [71]. For the experiment, the culture system was inoculated with 2.5 × 10^7^ AcH 505 spores at 3 and 7 weeks after establishment of the microcosm. With this form of inoculation, AcH 505 stimulates oak-*P. croceum* mycorrhiza formation [25], and elicits plant defences in leaves against the powdery mildew *Microsphaera alphitoides* [26]. After 4 weeks, 5 ml of soil filtrate was added to each culture system to establish a natural microbial community and thereby making the system closer to natural conditions. Soil filtrates were prepared as described by Rosenberg, et al. [72]. Briefly, the microbial filtrate was obtained by suspending the same soil from an oak forest stand that was used as a soil substrate for the microcosms in water and filtering it through 5.0 and 1.2 μm paper filters, thereby excluding Protozoa.

The oak microcuttings were cultivated for 8 weeks in climate chambers at 23 °C with a 16/8 h day/night (photosynthetic photon flux density of 180 μmol m^−2^ s^−1^ ), before the tissues were harvested. After harvest, tissues were immediately submerged in liquid nitrogen and stored at −80 °C. Harvest times were based on published effects of AcH 505 on EM formation and plant growth [25, 26].

For our investigations we only used plants that were in the developmental root flush (RF) and shoot flush (SF) stages, corresponding respectively to bud swelling stage B and leaf expansion stage D according to Herrmann, et al. [18]. The experimental design resulted in six treatments: 3 (no inoculation/AcH 505/AcH 505 + *P. croceum*) × 2 (RF/SF).

### RNA extraction and transcript quantitation by Illumina sequencing

For transcriptomic analyses, lateral roots and leaves were used. Source and sink leaves were used for plants during RF and SF respectively. Samples from 2–3 plants subjected to each treatment were pooled and homogenized under liquid nitrogen. Total RNA was isolated from 36 pools (3 treatments × 2 tissues × 2 developmental stages × 3 replicates) using the MasterPure Plant RNA Purification Kit (Epicentre, Hessisch Oldendorf, Germany) with 100 mg of root and 50 mg of leaf material per extraction. RNA quality and quantity were verified using gel electrophoresis, a NanoDrop 1000 spectrophotometer and an Agilent 2100 Bioanalyzer prior to Illumina sequencing analysis at the Beijing Genomics Institute (Hong Kong, China). 100 bp paired-end Illumina Truseq version 2 libraries were constructed and sequenced using the Illumina HiSeq2000 sequencing platform, and the sequence data was deposited as fastq files to the NCBI Short Read Archive, linked to a report specific BioProject termed PRJNA280092.

### Read processing and analysis of differential expression

Reads were processed following Tarkka, et al. [30]. Briefly, low quality sequences and sequencing artefacts were removed with SeqClean (http://sourceforge.net/projects/seqclean/files/) and low quality sequencing ends were trimmed with a custom Java script. Short sequences (<50 bp) and sequences lacking paired-end information were discarded. The processed Illumina reads were aligned against the reference transcriptome OakContigDF159.1 [30] by Bowtie [73] and quantified by RSEM [74]. Fold-changes in gene expression were calculated by pairwise comparisons using the edgeR function [75] implemented in the Bioconductor package [76]. In these comparisons, negative binomial models are fitted to the transcript abundancies determined by RSEM. Contigs were considered differentially expressed (DE) when the Benjamini-Hochberg adjusted P-value of this fit was less than 1 %. Blast2GO was used to get a description for each contig based on up to 20 hits against NCBI NR database in a blastx search (E-value 1e-5). Protein sequences from *Arabidopsis thaliana* TAIR database were downloaded to perform a blastx search of DF159.1 and to assign homologue proteins from *A. thaliana* to each contig. Only hits with an E-value of at least 1e-5 were taken into account. The best *A. thaliana* protein hit for each oak contig was determined by taking the *A. thaliana* protein exhibiting the largest percent identity to the contig in the local alignment.

Gene Ontology (GO) [77] enrichment analysis was performed with the Bioconductor package GOseq [78]. GOseq performs a statistical test based on a hypergeometric distribution to determine if in a given list of DE tags (e.g. genes or contigs) tags assigned to a certain category (e.g. GO terms) are significantly enriched, i.e. if they occur more frequently than expected by chance. Thereby GOseq adjusts the estimation of the P-value for tag-length. We used the capability of GOseq to perform enrichment analyses for a second type of categories, Protein families (Pfam) [79]. The OakContigDF159.1 reference library, GO annotations as well as best blast hits of each contig have been deposited at www.trophinoak.de.

### Real-time-quantitative reverse transcriptase-PCR (qRT-PCR) primer design and reactions

Differential gene expression data obtained from the Illumina analyses were validated by qRT-PCR analysis of the expression of 10 genes in leaf samples from control and co-inoculated plants. Primer pairs were constructed using the OakContigDF159.1 assembly as a reference and tested for functionality, amplicon size, specificity and efficiency as previously described [30]. Sequences of constructed primer pairs are listed in Additional file 7. The qRT-PCR reactions were performed as described by Tarkka, et al. [30]. Briefly, using an iScript One-Step RT-PCR Kit with SYBR Green (Bio-Rad) and *18S rRNA* as the reference gene, transcript abundances in the leaf samples were determined based on their Ct values using the Relative Expression Software Tool [REST, 80]. The coefficient of variation was used as a reproducibility indicator, with a maximal value of 6.0. Differential gene expression was determined by a randomisation test implemented in REST.

## Availability of supporting data

Supporting data are included as Additional files.

The OakContigDF159.1 reference library, GO annotations as well as best blast hits of each contig have been deposited at www.trophinoak.de.

The sequence data as original fastq files is deposited at NCBI Short Read Archive, linked to a report specific BioProject termed PRJNA280092 (http://www.ncbi.nlm.nih.gov/bioproject/PRJNA280092).

## Additional files

Additional file 1:
**Results of Illumina sequencing of Quercus robur DF159 cDNA libraries.** (XLS 50 kb) Additional file 2:
**Mapping of reads on the OakContigDF159.1 reference transcriptome.** (DOCX 72 kb) Additional file 3:
**Results of** **qRT-PCR** **analysis.** (DOCX 16 kb) Additional file 4:
**Differential gene expression levels in numbers.** (DOCX 18 kb) Additional file 5:
**The most significantly enriched Pfam and GO terms.** (XLSX 31 kb) Additional file 6:
**DECs associated with enriched Pfam and GO terms.** (XLSX 57 kb) Additional file 7:
**Quantitative polymerase chain reaction primer sequences.** (DOCX 15 kb)

## Abbreviations

ABA: Abscisic acid
bZIP: Basic-domain leucine-zipper
CHLBP: Chlorophyll A-B binding protein
DE: Differentially expressed
DEC: Differentially expressed contig
EDC: Enriched in down-regulated contigs
EM: Ectomycorrhiza
EMF: Ectomycorrhizal fungus
ERF: Ethylene-responsive element binding factor
ET: Ethylene
EUC: Enriched in up-regulated contigs
FDA: Fasciclin domain arabinogalactan protein
GDSL: GDSL-like Lipase/Acylhydrolase
GO: Gene Ontology
JA: Jasmonic acid
LHCB: Light-harvesting chlorophyll a/b-binding protein
LRR: Leucine rich repeat
LRR-NB: Leucine rich repeat-nuleotide binding
LTP: Probable lipid transfer protein
MHB: Mycorrhiza helper bacterium
Pfam: Protein family
PIP1: Phosphate-induced protein 1
qRT-PCR: Real-time-quantitative reverse transcriptase-PCR
RF: Root flush
RLK: Receptor-like kinase
RLP: Receptor-like protein
SF: Shoot flush
TF: Transcription factor
SA: Salicylic acid
