# Supplementary material for: Large scale transcriptome analysis reveals interplay between development of forest trees and a beneficial mycorrhiza helper bacterium
Source: BMC Genomics. 2015 Sep 2;16(1):658. doi: 10.1186/s12864-015-1856-y (PMC4557895; doi:10.1186/s12864-015-1856-y)

**Additional file 2** Mapping of reads on the OakContigDF159.1 reference transcriptome. Reads were mapped against the OakContigDF159.1 assembly, and the number of contigs (y-axis) was plotted against the level of mapped reads (x-axis). The x-axis was subdivided into classes. Class 0 includes the contigs with no expression, and the other classes x contain all contigs with an expression level in (x-1, x], and finally the class "more" all contigs with an expression level higher than the maximal class value. Each sample is illustrated with dot of a specific color. Mapping of reads are shown both as raw counts (without normalisation), and as expression levels normalised by sequencing depth and contig length (FPKM). Note that the reads originating from different libraries have similar levels of mapping against OakContigDF159.1 reference transcriptome.


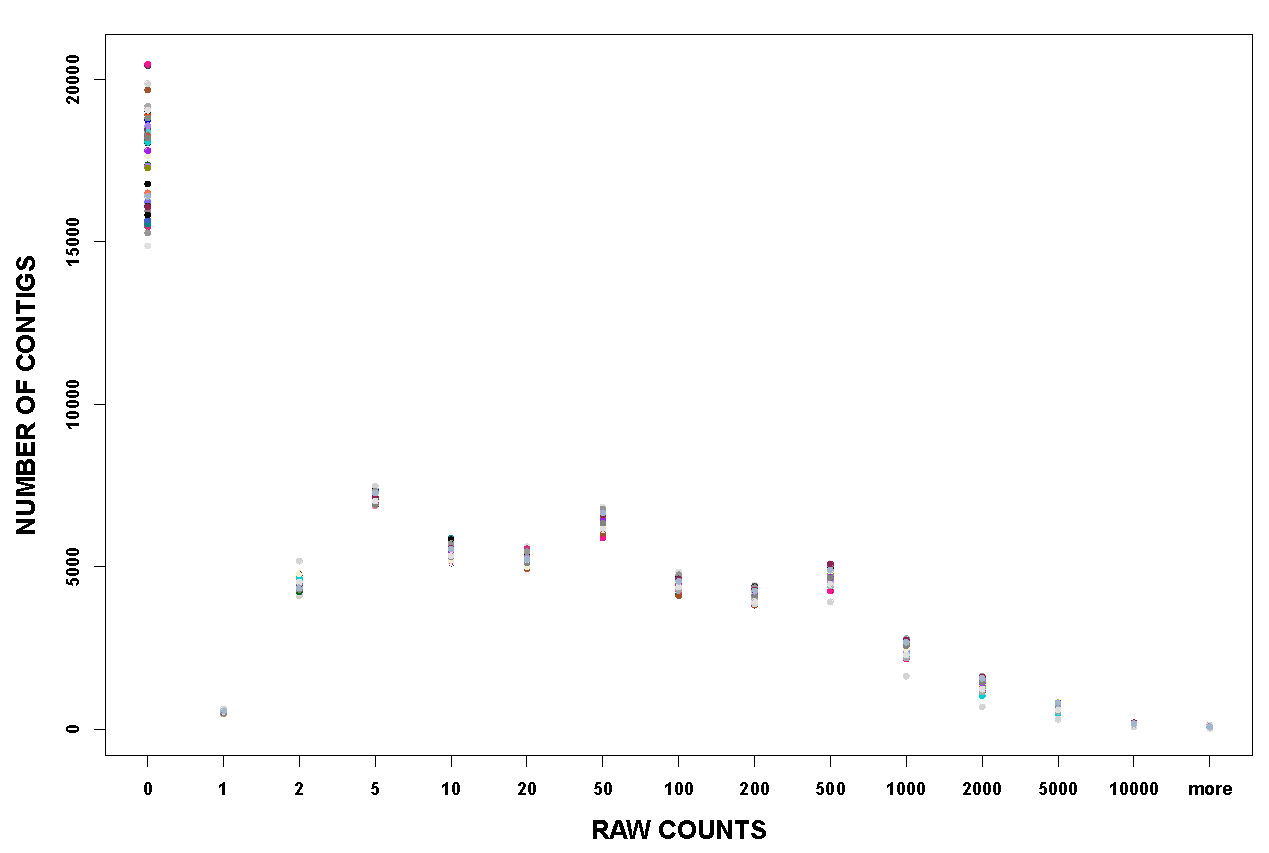


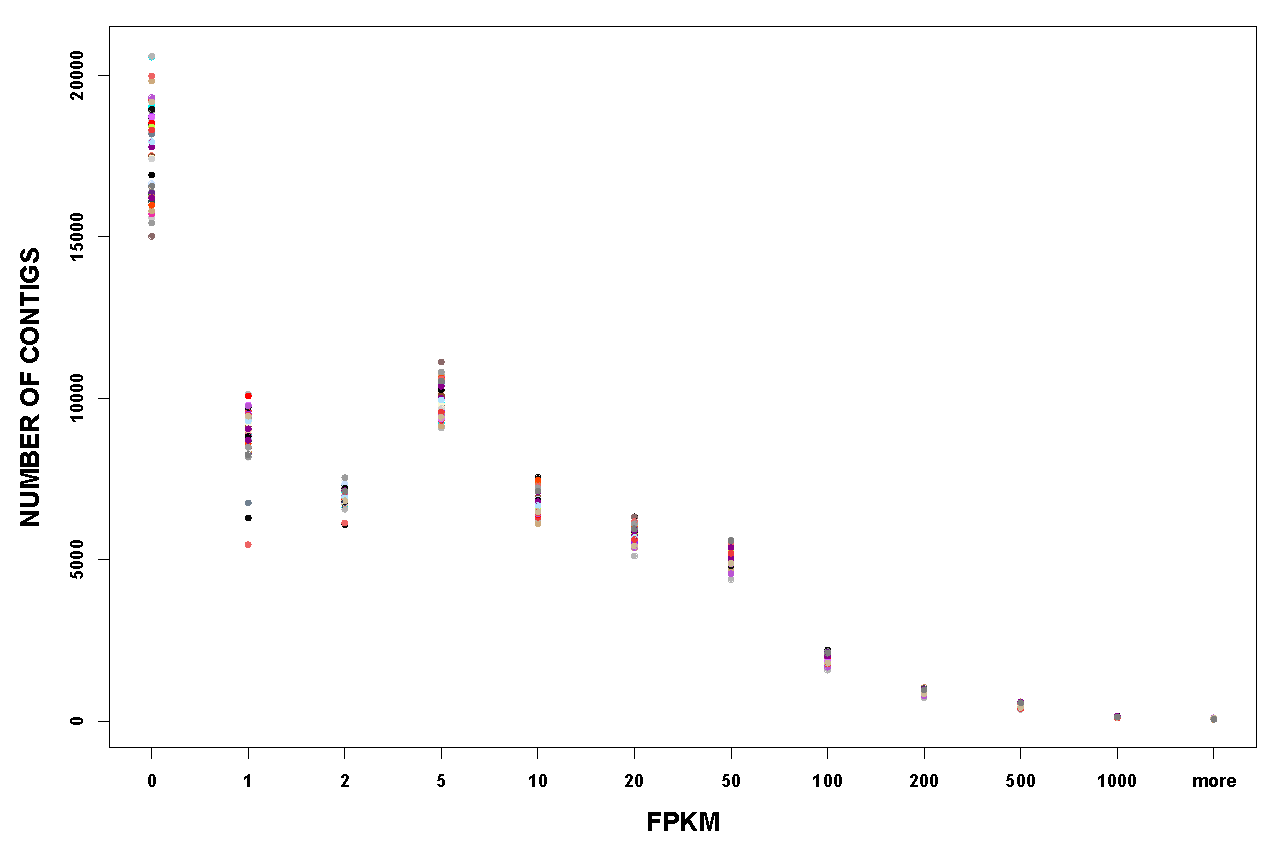

Supplement: Additional file 2: — Mapping of reads on the OakContigDF159.1 reference transcriptome. (DOCX 72 kb) [file 12864_2015_1856_MOESM2_ESM.docx]
