# Supplementary material for: Large scale transcriptome analysis reveals interplay between development of forest trees and a beneficial mycorrhiza helper bacterium
Source: BMC Genomics. 2015 Sep 2;16(1):658. doi: 10.1186/s12864-015-1856-y (PMC4557895; doi:10.1186/s12864-015-1856-y)
Supplement: Additional file 3: — Results of qRT-PCR analysis. (DOCX 16 kb) [file 12864_2015_1856_MOESM3_ESM.docx]

**Additional file 3** Results of qRT-PCR analysis of 10 contigs with increased or decreased expression levels in leaves of AcH 505 and *P. croceum-*inoculated seedlings relative to levels in Controls according to RNA-Seq.

| **Contig** | **RNA-Seq** | |  | **qRT-PCR** | | **Blastx predicted transcript identity** |
| --- | --- | --- | --- | --- | --- | --- |
|  | log2 fold change | p-value |  | log2 fold change | p-value |  |
| 39043 | -1.8 | 1.0E-02 |  | -1.0 | 3.5E-03 | Inositol oxygenase |
| 43608 | -1.7 | 4.0E-02 |  | -1.1 | 6.4E-02 | Pectinesterase |
| 43258 | -1.3 | 1.4E-03 |  | -1.1 | 8.0E-02 | Glutamate dehydrogenase |
| 43229 | -1.0 | 4.9E-03 |  | -2.0 | 6.6E-02 | Tata box binding protein associated factor |
| 34707 | -0.9 | 0.0E+00 |  | -0.3 | 4.6E-01 | Trehalose-phosphate synthase |
| 42379 | -0.9 | 2.0E-02 |  | -0.3 | 7.0E-01 | Phosphate transporter |
| 32318 | 0.7 | 4.0E-02 |  | 0.2 | 2.0E-02 | Calcium-binding allergen |
| 32511 | 1.1 | 1.6E-03 |  | 0.9 | 1.0E-03 | Phosphate transporter |
| 42037 | 1.2 | 1.3E-03 |  | 1.9 | 3.4E-02 | Peptide nitrate transporter |
| 39751 | 2.1 | 1.1E-03 |  | 0.3 | 7.4E-01 | Ap2 erf domain-containing transcription factor |
