# Supplementary material for: Large scale transcriptome analysis reveals interplay between development of forest trees and a beneficial mycorrhiza helper bacterium
Source: BMC Genomics. 2015 Sep 2;16(1):658. doi: 10.1186/s12864-015-1856-y (PMC4557895; doi:10.1186/s12864-015-1856-y)
Supplement: Additional file 4: — Differential gene expression levels in numbers. (DOCX 18 kb) [file 12864_2015_1856_MOESM4_ESM.docx]

**Additional file 4** Differential gene expression levels in numbers. Numbers and relative amounts of differentially expressed contigs detected in the pairwise comparisons of roots and leaves of Controls versus AcH 505-inoculated plants (Co-Ac) and Controls versus AcH 505- and *P. croceum*-inoculated plants during root flush (RF) and shoot flush (SF). Up- and down-regulated indicates up- and down-regulation in the inoculated plants, respectively (Benjamini-Hochberg adjusted p ≤ 0.01). The numbers of DECs, and the relative extent of the DECs, of the 64780 contigs of the OakContigDF159.1 reference transcriptome, are indicated.

| Numbers of differentially expressed contigs | | | | | |
| --- | --- | --- | --- | --- | --- |
|  | Root | | Leaf | | |
|  | Co-Ac | Co-AcPi | | Co-Ac | Co-AcPi |
| RF |  |  | |  |  |
| total | 182 | 469 | | 337 | 284 |
| up-regulated | 113 | 264 | | 226 | 139 |
| down-regulated | 69 | 205 | | 111 | 145 |
| SF |  |  | |  |  |
| total | 3490 | 593 | | 3043 | 193 |
| up-regulated | 1753 | 162 | | 1499 | 115 |
| down-regulated | 1737 | 431 | | 1544 | 78 |

| Percentages of differentially expressed contigs of the OakContigDF159.1 reference transcriptome | | | | | |
| --- | --- | --- | --- | --- | --- |
|  | Root | | Leaf | | |
|  | Co-Ac | Co-AcPi | | Co-Ac | Co-AcPi |
| RF |  |  | |  |  |
| total | 0.28 | 0.71 | | 0.51 | 0.43 |
| up-regulated | 0.17 | 0.40 | | 0.34 | 0.11 |
| down-regulated | 0.11 | 0.31 | | 0.17 | 0.22 |
| SF |  |  | |  |  |
| total | 5.3 | 0.90 | | 4.6 | 0.29 |
| up-regulated | 2.7 | 1.2 | | 2.3 | 0.17 |
| down-regulated | 2.6 | 0.66 | | 2.3 | 0.12 |
