# Supplementary material for: Large scale transcriptome analysis reveals interplay between development of forest trees and a beneficial mycorrhiza helper bacterium
Source: BMC Genomics. 2015 Sep 2;16(1):658. doi: 10.1186/s12864-015-1856-y (PMC4557895; doi:10.1186/s12864-015-1856-y)
Supplement: Additional file 7: — Quantitative polymerase chain reaction primer sequences. (DOCX 15 kb) [file 12864_2015_1856_MOESM7_ESM.docx]

**Additional file 7** Quantitative polymerase chain reaction primers. Blastx searches against the NCBI nr database were used to predict the identity of the target transcripts.

| **Primer** | **Sequence (5'-3')** | **Blastx predicted transcript identity** |
| --- | --- | --- |
| comp32318-f | CGGAAAGATAACGGCAGAGGA | Calcium-binding allergen |
| comp32318-r | GGAAGCACACGAACCCAT |  |
| comp32511-f | CTTATCTCCAAGCCACCCGA | Phosphate transporter |
| comp32511-r | GCCTCCCAATGTTTCAGC |  |
| comp34707-f | GCCATCAGTTGTCTCCGTGT | Trehalose-phosphate synthase |
| comp34707-r | GTGCGAATCTTGGTCTTGCG |  |
| comp39043-f | CAGCACATTTTCCAGTCCAC | Inositol oxygenase |
| comp39043-r | ACGCATCCTGTTGGTTGT |  |
| comp39751-f | GTCGCCGTTATCTCCTCAC | Ap2 erf domain-containing transcription factor |
| comp39751-r | GCCTCATCCACTCACACAC |  |
| comp42037-f | TTGGTGGCTTTAGGGAGTGG | Peptide nitrate transporter |
| comp42037-r | GAGTGTCTTGGATTGCTTGGAG |  |
| comp42379-f | TGGATAACTCCTCCCTTGG | Phosphate transporter |
| comp42379-r | TCTTTCCCTCATTGCCTTGG |  |
| comp43229-f | CCTCCTTCGCCCTTATCTGC | Tata box binding protein associated factor |
| comp43229-r | ACTGTGACTTGAGCCCTGTG |  |
| comp43258-f | GCGGGGAGTGTAAGAATAGC | Glutamate dehydrogenase |
| comp43258-r | AGGGGTTGGGAAGCATAA |  |
| comp43608-f | TGCTTGATGGGGTTGTGGTA | Pectinesterase |
| comp43608-r | GTGCCTCTTTTTCTGTCTCC |  |
